# Supplementary material for: Molecular switching in transcription through splicing and proline-isomerization regulates stress responses in plants
Source: Nat Commun. 2024 Jan 18;15:592. doi: 10.1038/s41467-024-44859-2 (PMC10796322; doi:10.1038/s41467-024-44859-2)
Supplement: Supplementary file 1 — Supplementary Information [file 41467_2024_44859_MOESM1_ESM.pdf]

Molecular switching in transcription through splicing and proline-isomerization regulates stress responses in plants

Frederik Friis Theisen[1,2], Andreas Prestel[2], Steffie Elkjær[1], Yannick H. A. Leurs[1], Nicholas Morffy[3], Lucia C. Strader[3], Charlotte O’Shea[1], Kaare Teilum[2], Birthe B. Kragelund[1,2]* and Karen Skriver[1]*

[1]The REPIN and The Linderstrøm-Lang Centre for Protein Science, Department of Biology, University of Copenhagen, Copenhagen, Denmark; [2]Structural Biology and NMR Laboratory, Department of Biology, University of Copenhagen, Copenhagen, Denmark; [3]Department of Biology, Duke University, Durham, USA.

*Corresponding authors: bbk@bio.ku.dk; kskriver@bio.ku.dk.

**Residue/Peak**	**V_{trans}**	**V_{cis}**	**V_{total}**	**$\%_{trans}$**	**$\%_{cis}$**
**25 °C**					
Asp241 ^{15}N,H^{N}	7552	3042	10594	71.3%	28.7%
Gly243 ^{15}N,H^{N}	6628	2748	9375	70.7%	29.3%
Trp244 ^{15}N,H^{N}	7591	2991	10582	71.7%	28.3%
Gln250 ^{15}N,H^{N}	7201	3077	10278	70.1%	29.9%
Trp244 ^{15}N$^{\varepsilon}$,H$^{\varepsilon}$	6051	2796	8846	68.4%	31.6%
Mean				70.4%	29.6%
SD				1.3%	1.3%
**20 °C**					
Asp241 ^{15}N,H^{N}	8092	3300	11391	71.0%	29.0%
Gly243 ^{15}N,H^{N}	7019	2912	9931	70.7%	29.3%
Trp244 ^{15}N,H^{N}	8071	3226	11297	71.4%	28.6%
Gln250 ^{15}N,H^{N}	7774	3496	11270	69.0%	31.0%
Trp244 ^{15}N$^{\varepsilon}$,H^{N}	7448	3062	10510	70.9%	29.1%
Mean				70.6%	29.4%
SD				0.9%	0.9%
**15 °C**					
Asp241 ^{15}N,H^{N}	8141	3296	11437	71.2%	28.8%
Gly243 ^{15}N,H^{N}	7031	2912	9942	70.7%	29.3%
Trp244 ^{15}N,H^{N}	8041	3217	11257	71.4%	28.6%
Gln250 ^{15}N,H^{N}	7462	3338	10800	69.1%	30.9%
Trp244 ^{15}N$^{\varepsilon}$,H^{N}	7538	3126	10664	70.7%	29.3%
Mean				70.6%	29.4%
SD				0.9%	0.9%
**10 °C**					
Asp241 ^{15}N,H^{N}	8222	3351	11573	71.0%	29.0%
Gly243 ^{15}N,H^{N}	7081	2947	10028	70.6%	29.4%
Trp244 ^{15}N,H^{N}	8059	3327	11386	70.8%	29.2%
Gln250 ^{15}N,H^{N}	7554	3432	10986	68.8%	31.2%
Trp244 ^{15}N$^{\varepsilon}$,H^{N}	7583	3154	10737	70.6%	29.4%
Mean				70.4%	29.6%
SD				0.9%	0.9%

Supplementary Table 1: Unbound DREB2A *cis* and *trans* population HSQC peak volumes at different temperatures. ^{15}N,H^{N}-HSQC peak volumes (V) for five peaks which could be assigned in both *cis* and *trans* states. Volumes were determined by fitting lineshapes using PINT[1]. HSQCs (Supplementary Fig. 9) were recorded using a recycle delay of 6 seconds to reduce effects of potential relaxation differences between populations.

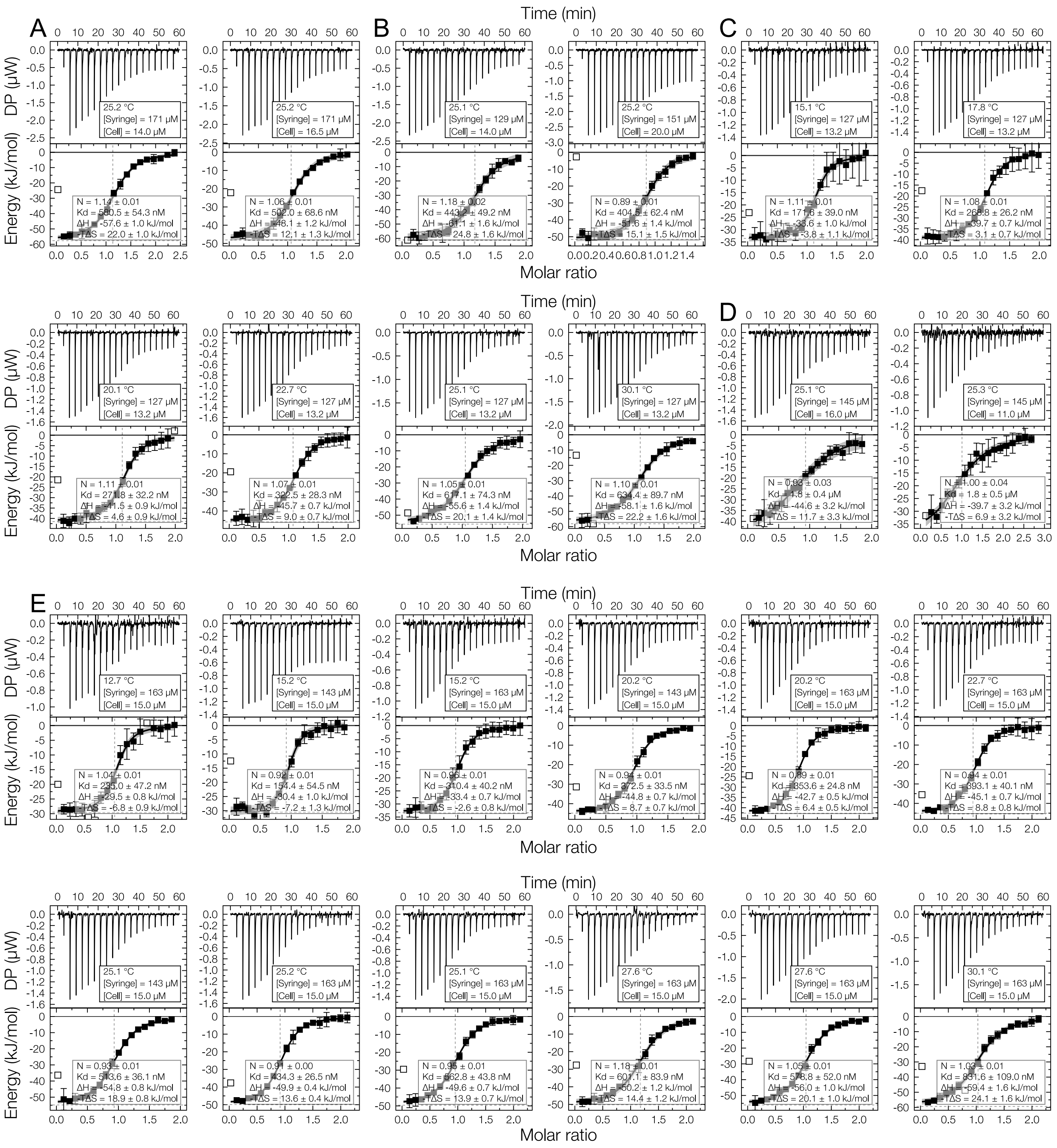

Supplementary Figure 1: Isothermal titration calorimetry data analysis. Analysis of Med25-ACID$_{532-680}$ ITC data used in truncation study. Top graphs show the baseline corrected data and bottom shows the one-set-of-sites model fit of integrated peaks. Experimental temperature and concentrations are given in each data plot and resulting fitting parameters are shown in the fitting plot. Error bars indicate differential power noise relative to peak area. Hollow symbols were not used in fitting. Vertical and horizontal dashed lines represent the N-value and the fitted enthalpy, respectively. From left to right: (A) DREB2A$_{151-335}$, (B) DREB2A$_{195-276}$, (C) DREB2A$_{234-335}$, (D) DREB2A$_{234-272}$, (E) DREB2A$_{234-276}$ (ABS-RIM), (F) (next page) DREB2A$_{234-256}$ (ABS), (G) DREB2A$_{255-276}$ (RIM), and (H) DREB2A$_{195-335,\Delta 244-276}$.

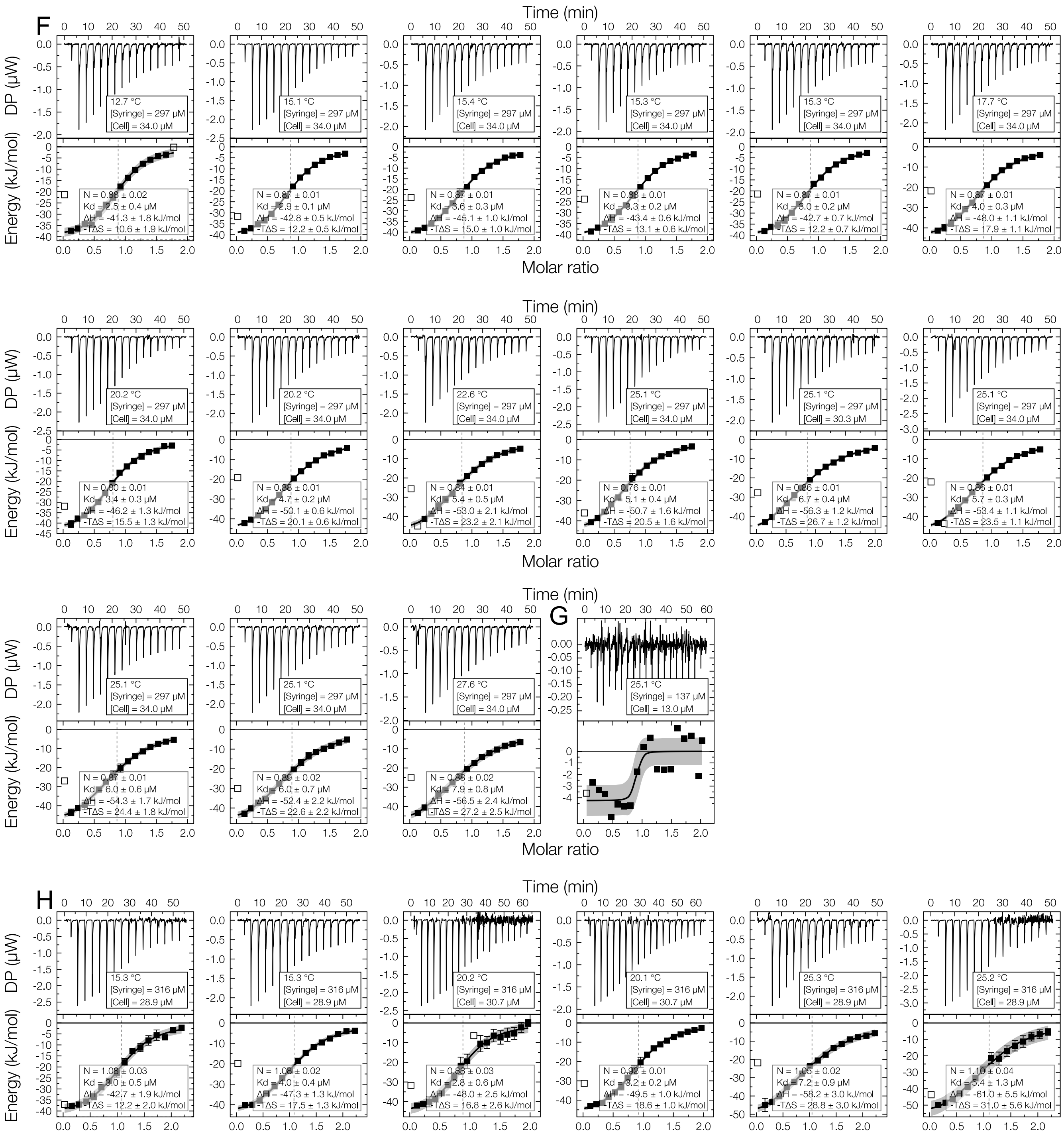

Supplementary Figure 1: Continued. Description on previous page.

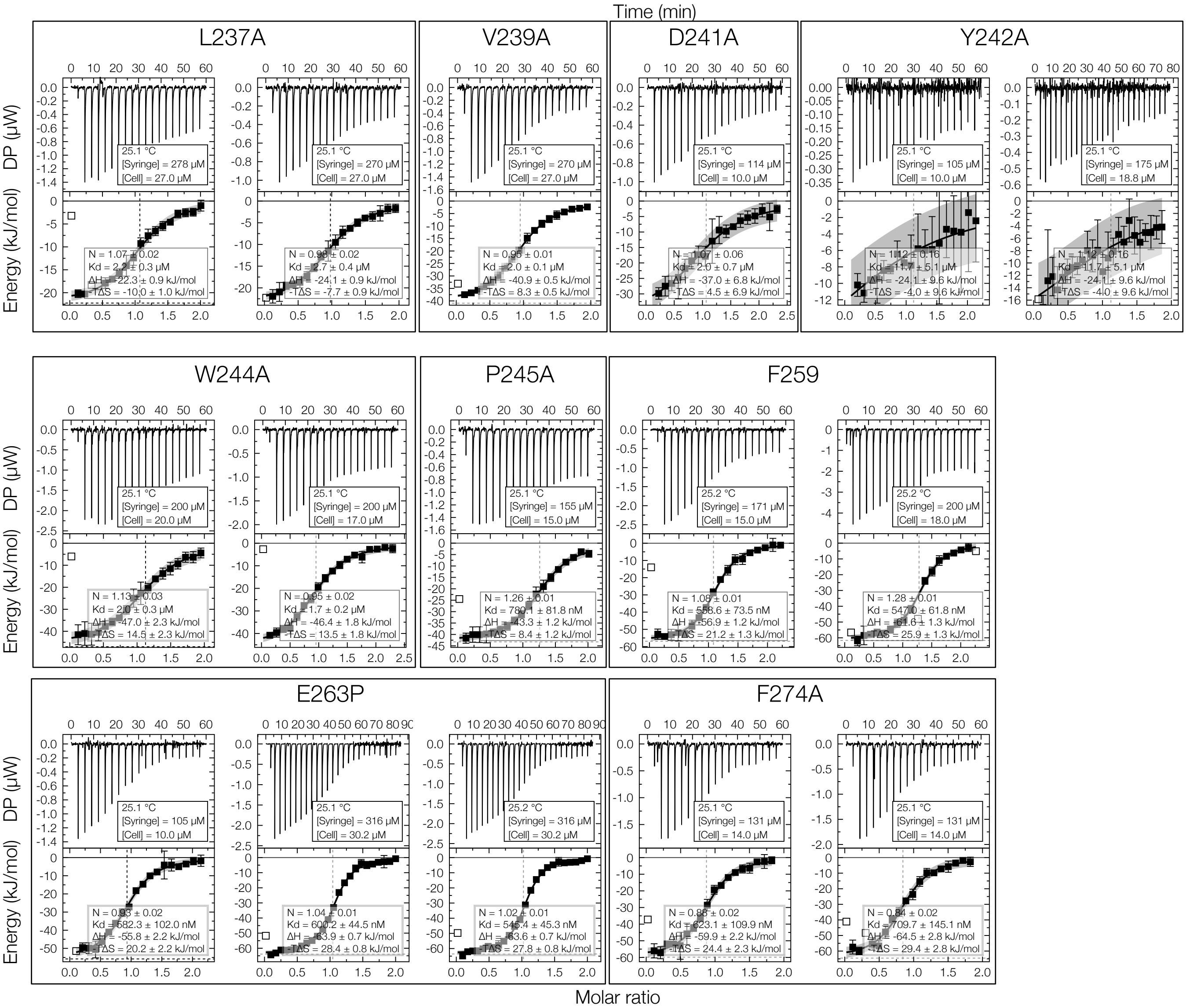

Supplementary Figure 2: Isothermal titration calorimetry of DREB2A variants. All experiments were done multiple times, but only those used for analysis are shown. Mutations were done in DREB2A$_{195-276}$ except for P245A which was done in DREB2A$_{234-276}$. The two Y242A experiments were fitted together using a global model, however fitted parameters are ambiguous. The specific variant is given above the isotherms. Error bars indicate differential power noise relative to peak area. Hollow symbols were not used in fitting.

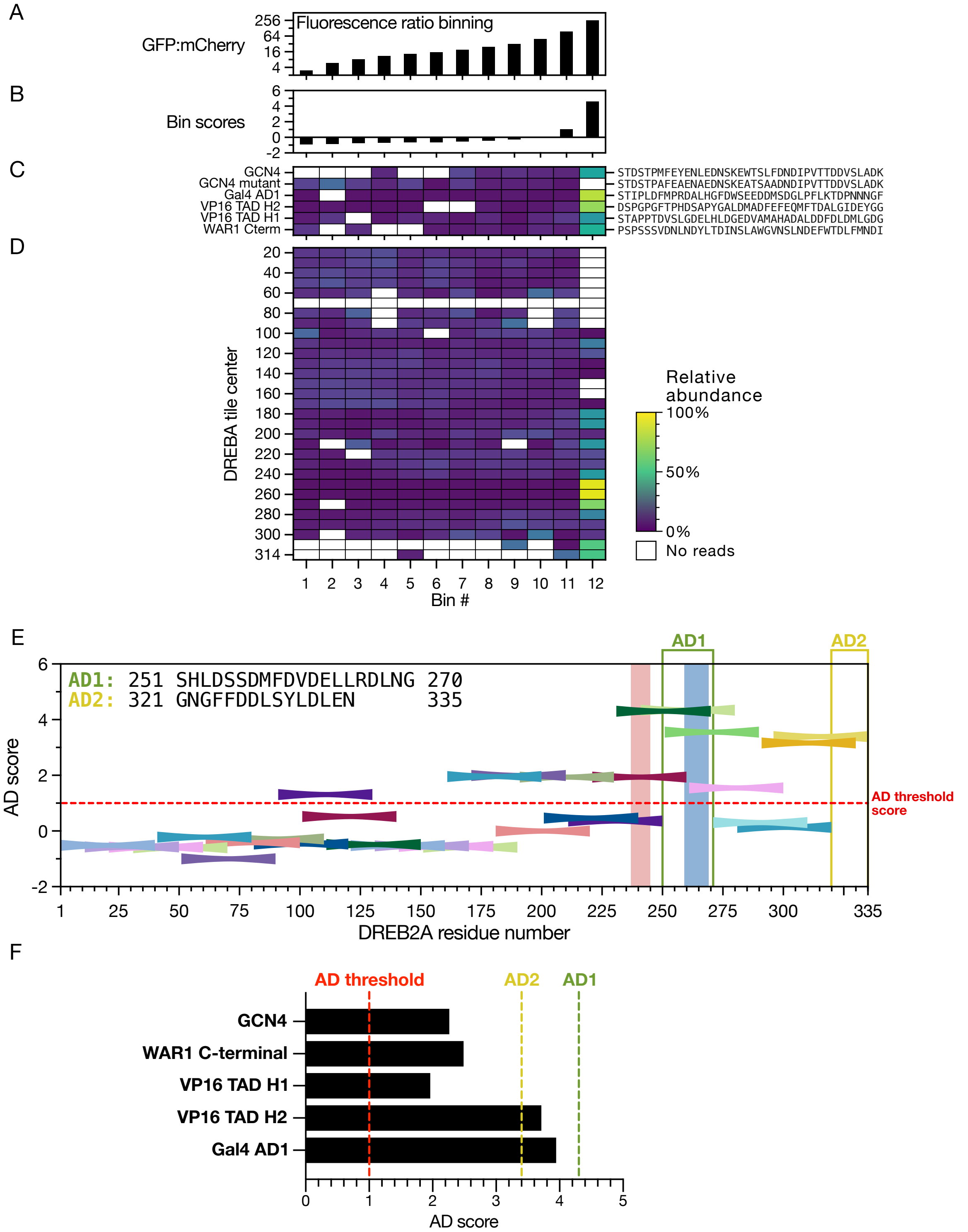

Supplementary Figure 3: Activation domain assay binning and individual DREB2A tile scores. (A) Activation domain assay fluorescence binning key. (B) Bin scores used to calculate the AD score. (C) AD normalized bin read counts for control ADs. (D) Fragment normalized bin read counts for all DREB2A tiles. Cells were sorted and binned by GFP:mCherry fluorescence ratio according to the levels given in panel A. (E) Individual 40-residue DREB2A tile AD scores calculated by summing the bin score (panel B) times bin abundance (panel D) product of each bin. Tiles are colored to distinguish individual tiles. ABS (red) and RIM (blue) regions are shown along with AD1 and AD2. Sequences of AD1 and AD2 are also given. (F) Activation domain assay scores for synthetic TFs containing known ADs. The maximum tile scores of the regions comprising AD1 and AD2 in DREB2A are shown.

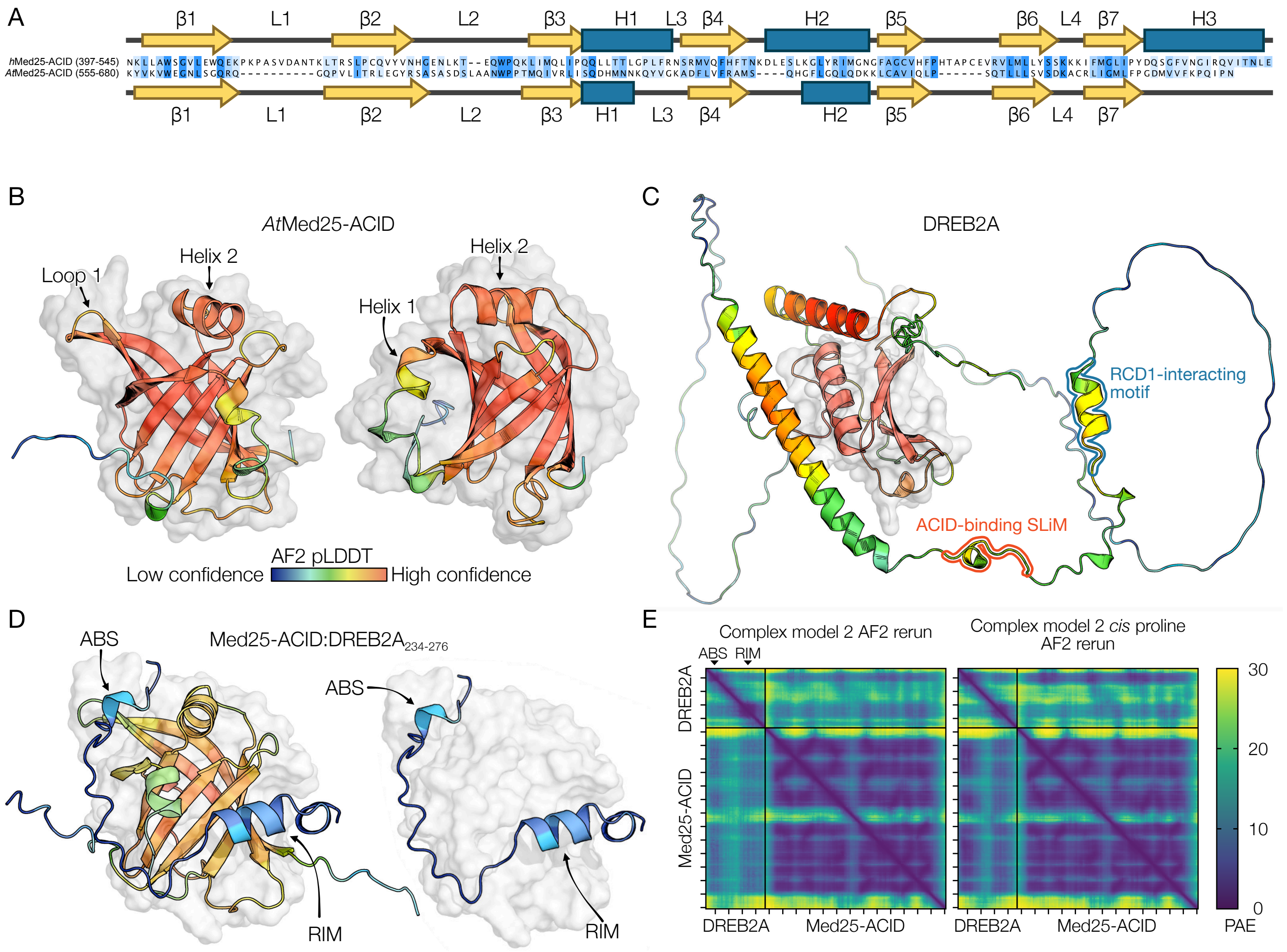

Supplementary Figure 4: Med25-ACID sequence alignment, AlphaFold2 predictions and confidence mapping. (A) Structure-based sequence alignment of *human* and *Arabidopsis thaliana* Med25-ACID. Secondary structure derived from PDB ID 2XNF (*human*) and AF2 model (*Arabidopsis*). AlphaFold2 predictions of (B) *At*Med25-ACID (Uniprot: Q7XYY2) (C) *At*DREB2A (Uniprot: O82132) colorized according to the pLDDT confidence score from the EMBL AF2 database. The ABS and RIM motifs are shown in red and blue, respectively. (D) ColabFold prediction of the Med25-ACID:DREB2A$_{234\text{-}276}$ complex structure. Shown with (left) and without (right) Med25-ACID cartoon representation. (E) AF2 predicted aligned errors (PAEs) of the ABS-RIM DREB2A fragment in complex with Med25-ACID obtained using the previously predicted model 2 as template. PAE matrices are shown for both *trans* and *cis* proline structures. The *cis* proline structure was generated using Gromacs by performing a 1 ns MD simulation using a backbone angle restraint to obtain the *cis* conformation. Yellow color indicates low confidence in the relative position of two residues. Values in the off-diagonal blocks provide an indication of complex structure confidence at specific positions.

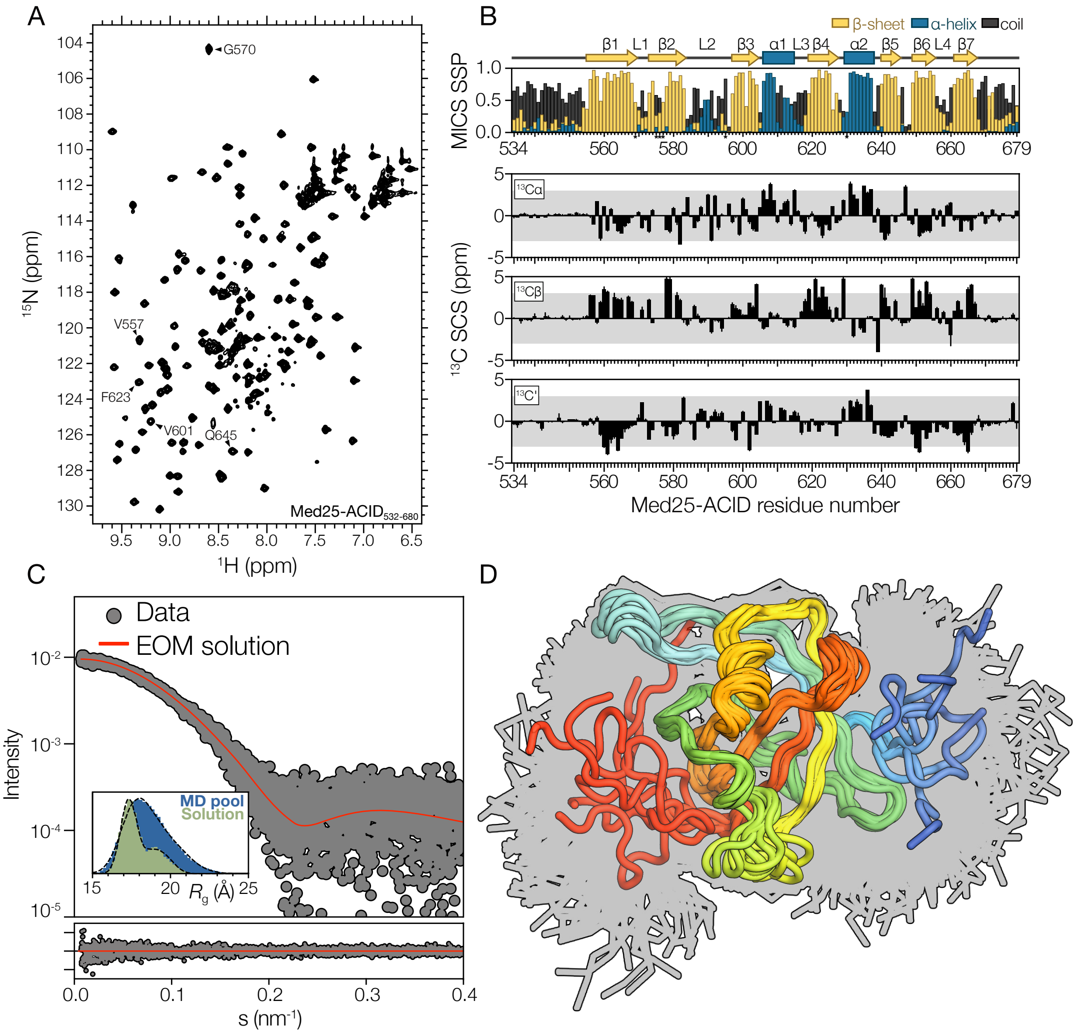

Supplementary Figure 5: Characterization of the *At*Med25-ACID domain structure using NMR and SAXS. (A) ^{15}N-HSQC spectrum of free Med25-ACID$_{532\text{-}680}$. (B) Med25-ACID $^{13}C^{\alpha}$, $^{13}C^{\beta}$, and ^{13}C' secondary chemical shifts calculated using the SBiNLab and POTENCI web tools. For $^{13}C^{\alpha}$ and ^{13}C', consecutive positive values indicate helical structure while consecutive negative values indicate extended structure. For $^{13}C^{\beta}$ the correlation is inverse. Values close to zero may indicate dynamic random coil structure. The grey bars represent ±3 ppm. The MICS analysis, which uses chemical shift data from all assigned nuclei to predict secondary structure, is shown above. (C) SAXS scattering data analyzed with EOM using a structure pool generated from a 1.7 µs MD simulation. The EOM solution ensemble scattering curve is shown in red and the residuals are shown below. (D) Structures from the EOM solution ensemble superimposed on the structure pool. The shown structures are truncated by 17 residues in the N-terminus (blue).

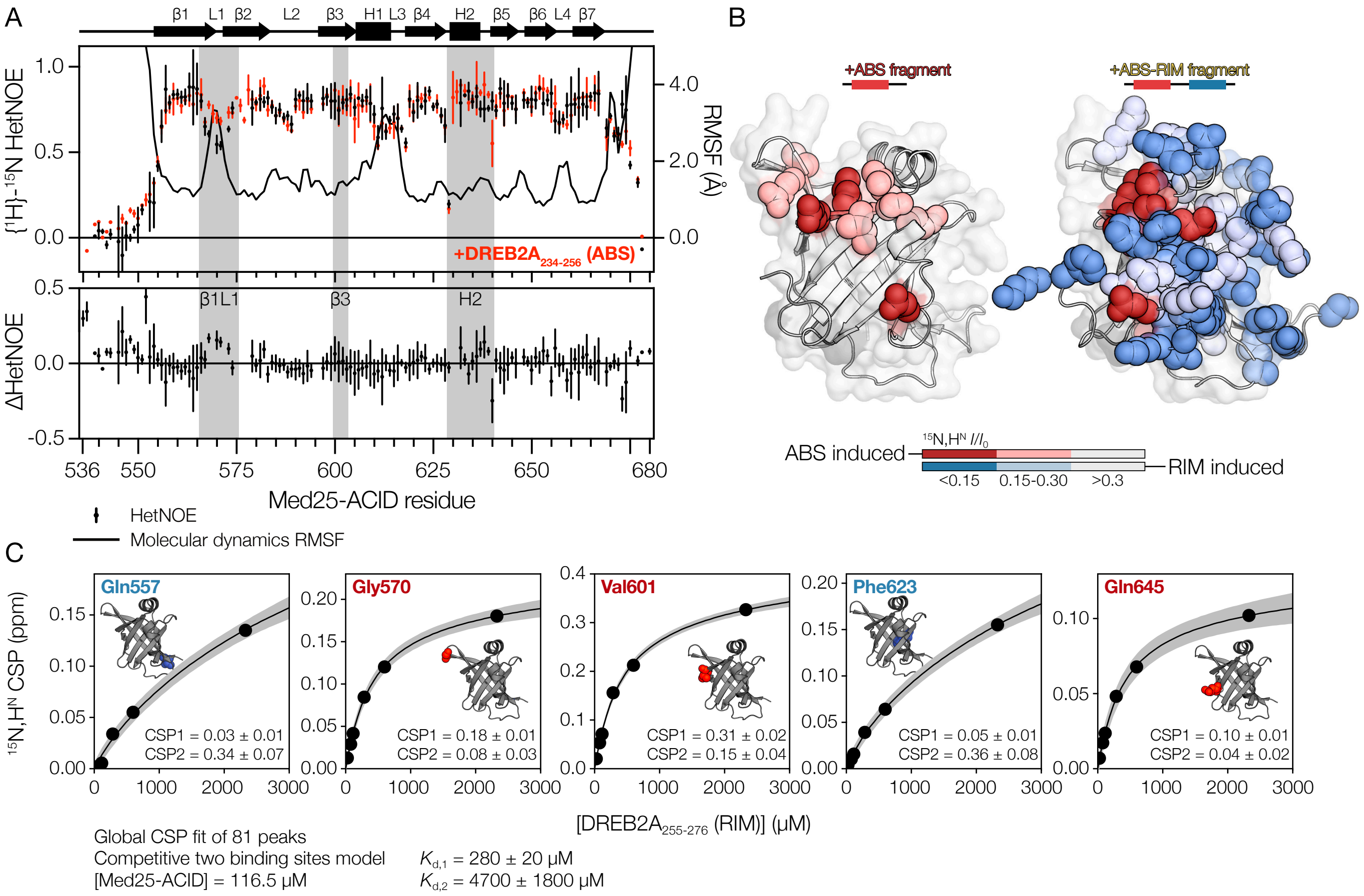

Supplementary Figure 6: Med25-ACID dynamics and peak intensity changes upon interaction with DREB2A. (A) {^{1}H}-^{15}N hetNOEs of bound Med25-ACID in complex with ABS (DREB2A$_{234-256}$) superimposed on the ratios obtained for unbound Med25-ACID. Differences are shown below. Grey boxes mark the structures defining the ABS-binding groove (residues within 4 Å of the ABS in AF2 model 2). The ACID domain consists of residues 555 to 675. (B) Mapped relative ^{15}N,H^{N}-HSQC peak intensities of Med25-ACID in complex with ABS (right) and ABS-RIM (left). Residues with relative intensities below 0.3 are colored and shown with spheres. Intensity loss induced by ABS binding is colored red, while ABS-RIM-induced additional effects are colored blue. (C) Global fit of 81 RIM (DREB2A$_{255-276}$) induced ^{15}N,H^{N} CSPs using a competitive binding model. Example residues from each of the five regions defining the two binding sites. Structures show the location of the observed residue. Error bands are 95% confidence intervals.

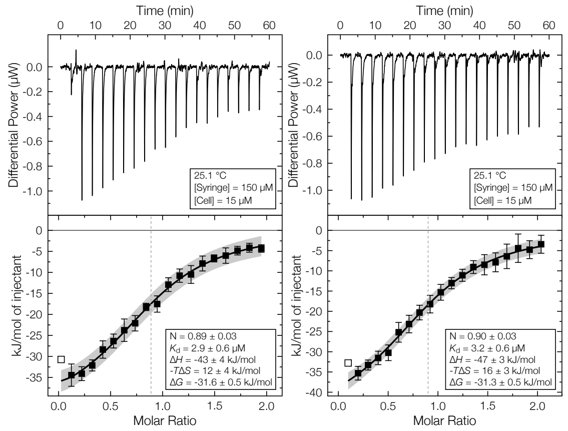

Supplementary Figure 7: Isothermal titration calorimetry results of the *At*Med25-ACID R568A variant. DREB2A$_{195-276}$ was used for the experiments. Error bars indicate baseline differential power noise relative to peak area. Hollow symbols were not used in fitting. The two panels show technical replicates.

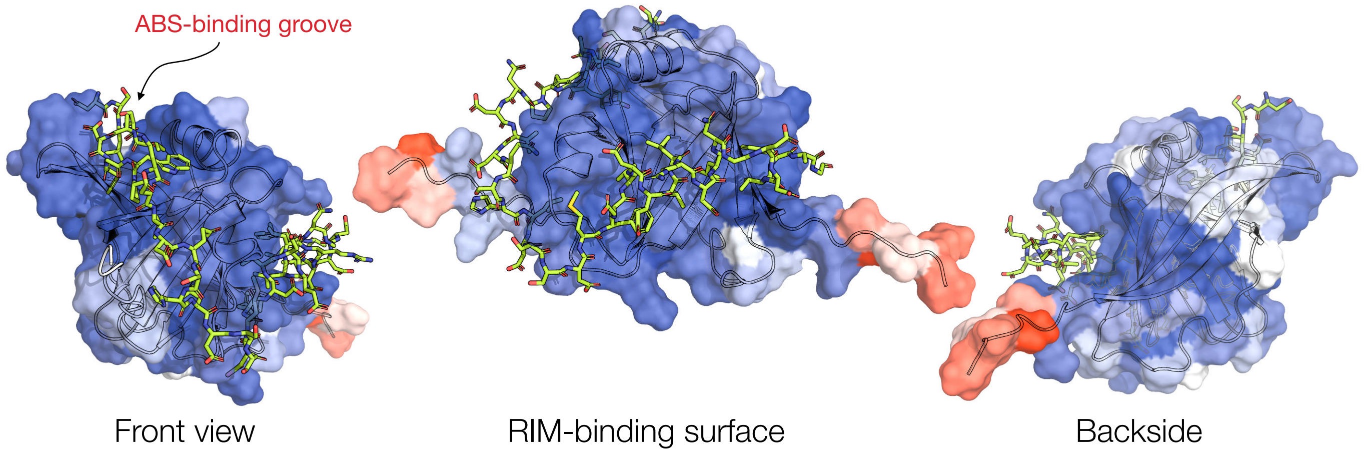

Supplementary Figure 8: *At*Med25-ACID sequence conservation mapped on the AF2 predicted complex structure. Conservation level is colored from red (low) through white to blue (high). The shown structure corresponds to AF2 complex model 2. DREB2A ABS-RIM is shown in green sticks.

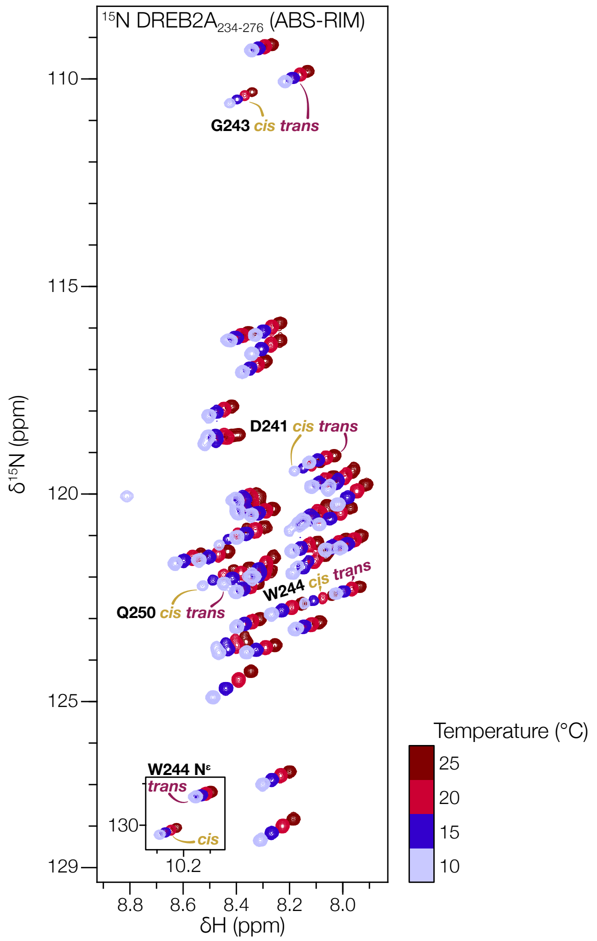

Supplementary Figure 9: Temperature titration of labeled DREB2A$_{234\text{-}276}$. ^{15}N,H^{N}-HSQC spectra of DREB2A$_{234\text{-}276}$ (ABS-RIM) in its free state, recorded in 5 °C intervals from 10 (light blue) to 25 °C (dark red). Residues used to determine *cis* and *trans* populations are highlighted and derived populations are shown in Supplementary Table 1.

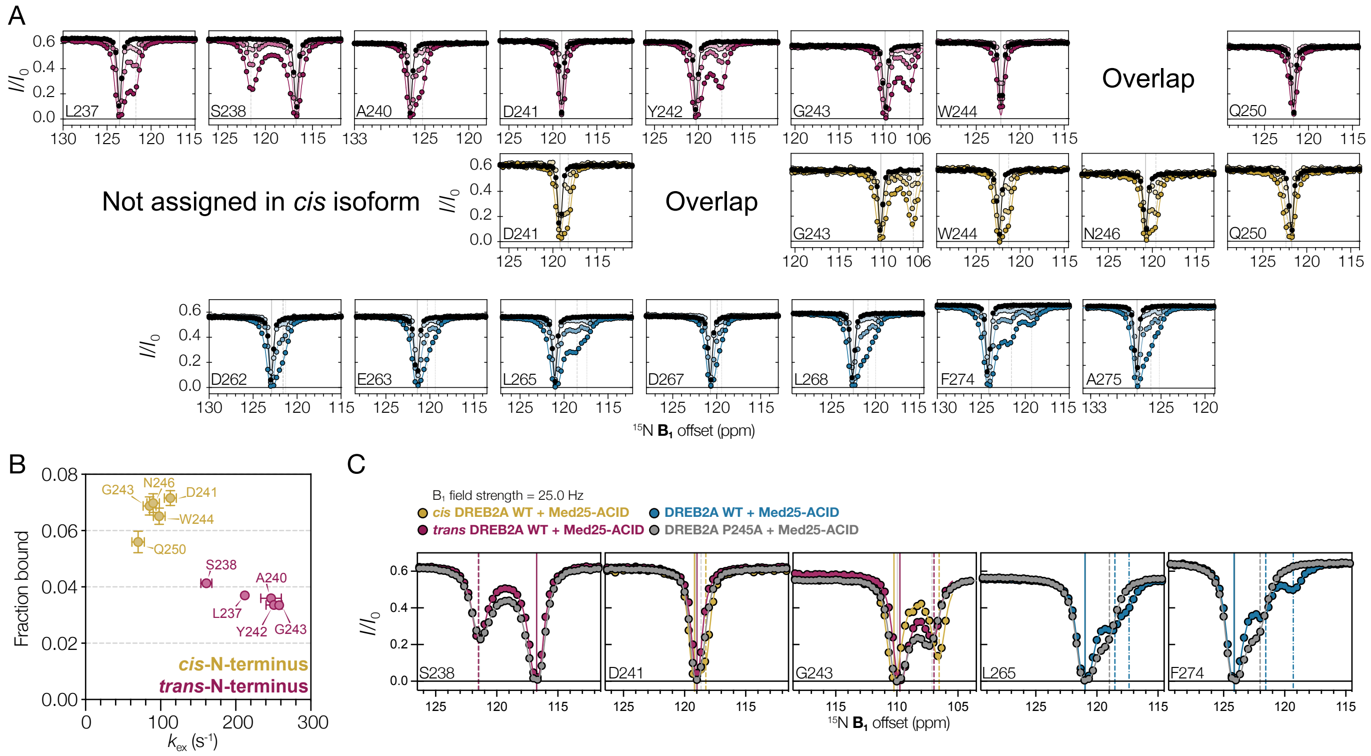

Supplementary Figure 10: ^{15}N-CEST profiles of DREB2A residues with 5% Med25-ACID. ^{15}N-CEST profiles of ^{13}C,^{15}N-labeled DREB2A$_{234\text{-}276}$ (ABS-RIM) with (colored) and without (black) 5% unlabeled Med25-ACID$_{532\text{-}680}$ recorded at 25 °C. Data without ligand was recorded using a $\mathbf{B_1}$ field strength of 12.5 Hz. For the profiles with Med25-ACID, three $\mathbf{B_1}$ field strengths (6.25, 12.5 and 25.0 Hz) were used. (A) Non-overlapping DREB2A CEST profiles of residues showing chemical exchange. Profiles from *trans* isoform residues Asp241, Trp244, and Gln250 are included for comparison with *cis* isoform profiles. but were not included in the global exchange model fitting. Residues N-terminal of Asp241 could not be assigned in the *cis* isoform or had too much overlap to analyze. Overlap labels indicate that the peak corresponding to the matching residue overlapped with other peaks in the HSQC, thus preventing quantitative analysis. C-terminus profiles exhibiting three-state behavior. (B) Individual analysis of N-terminal peaks showing distinct behavior of *cis* and *trans* populations. (C) Selected CEST profiles of the DREB2A P245A variant (grey), with corresponding WT profiles shown. Vertical lines indicate fitted chemical shifts of the ground state (solid) and minor states (dashed and dot dashed).

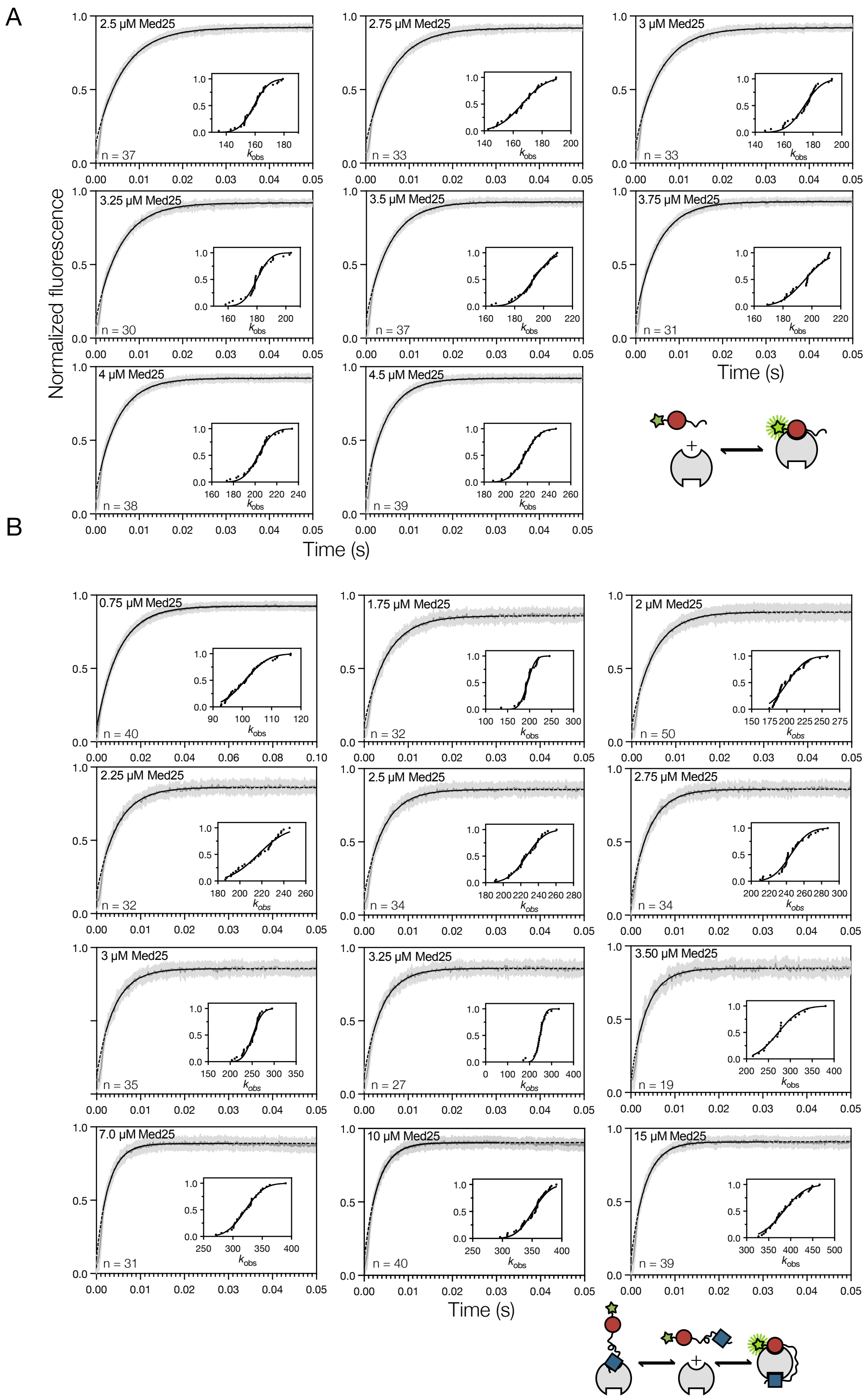

Supplementary Figure 11: Stopped-flow association kinetics of FITC-DRBE2A with Med25-ACID. FITC-DREB2A$_{234-256}$ (ABS) (A) and FITC-DREB2A$_{234-276}$ (ABS-RIM) (B) association data represented as normalized and averaged traces. Insets show individual trace fitting distributions of observed rate constants. All fluorescence traces were fitted using a single exponential decay function (solid line). Dashed line indicates extrapolate fit line. For FITC-DREB2A$_{234-276}$, three biological replicates of concentrations between 1.75 and 3.50 µM Med25-ACID were performed, but the differences between derived parameters were insignificant. *N* numbers refer to the number of technical replicates used for analysis.

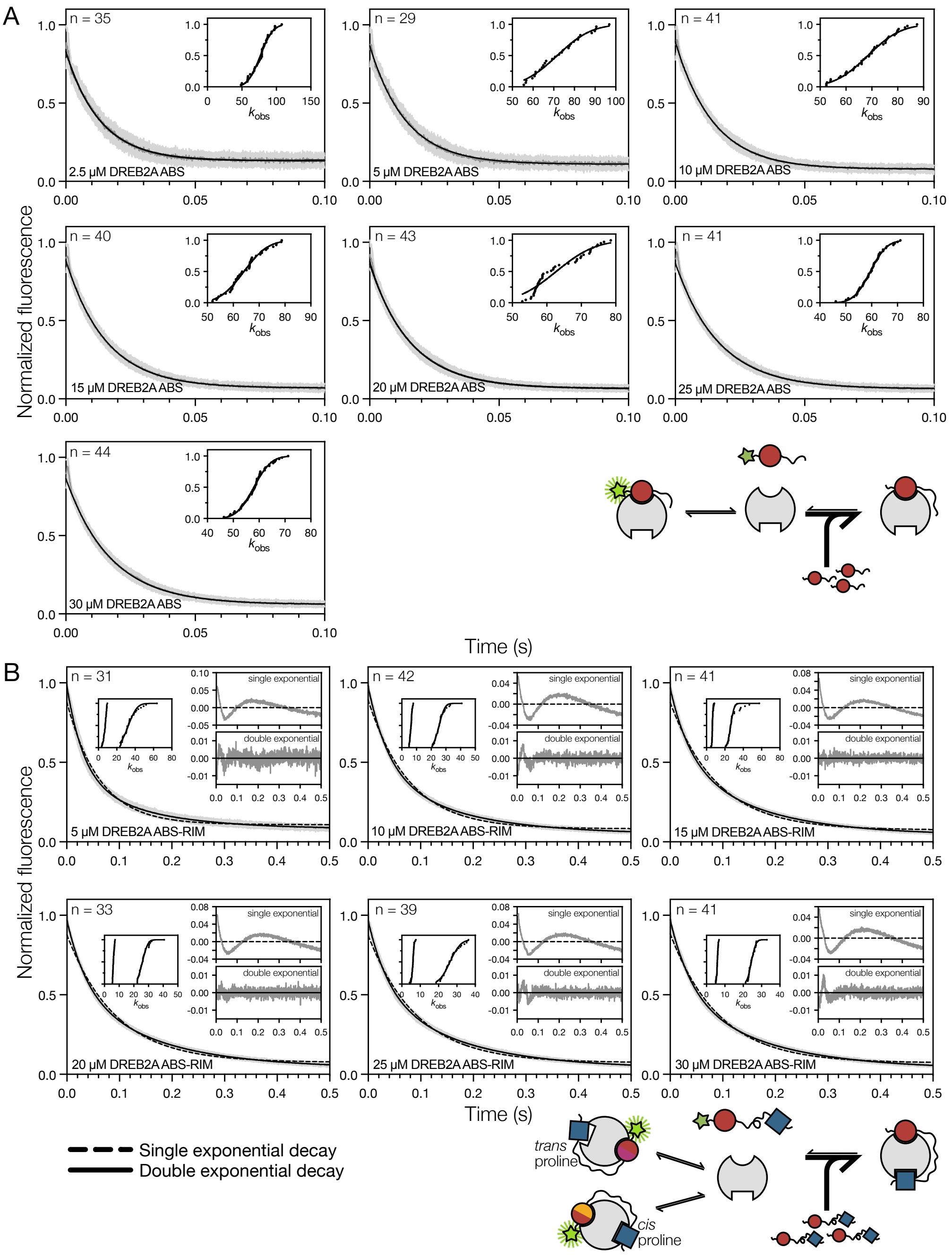

Supplementary Figure 12: Stopped-flow competitive displacement kinetics experiments of FITC-DREB2A and Med25-ACID. All fluorescence traces are represented as normalized and averaged traces. (A) FITC-DREB2A$_{234-256}$ (ABS) dissociation data with insets showing the distribution of individual trace fits. All traces fitted using a single exponential decay function. (B) FITC-DREB2A$_{234-276}$ (ABS-RIM) dissociation data, with insets showing double exponential decay derived observed rate constants and fit residuals of single (top, dashed line) and double (bottom, solid line) exponential decay functions. For both panel A and B, an inset is included showing the cumulative distribution of the observed rate constant(s). *N* numbers refer to the number of technical replicates used for analysis.

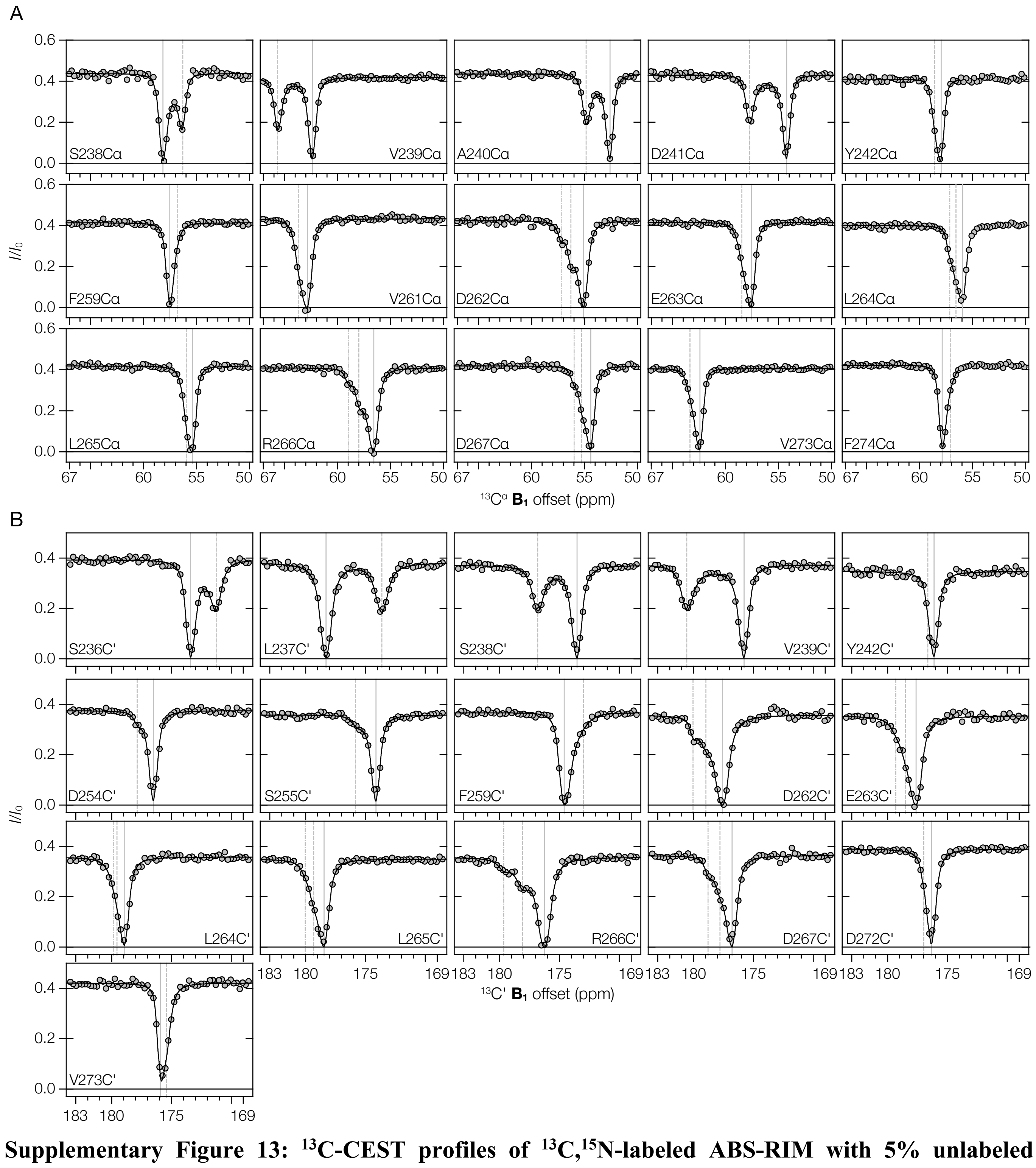

Supplementary Figure 13: ^{13}C-CEST profiles of ^{13}C,^{15}N-labeled ABS-RIM with 5% unlabeled Med25-ACID. The pulse sequences used modulate HSQC peaks as function of i-1 carbon saturation such that the HSQC peak corresponding to residue e.g. Tyr242 would be modulated as a function of Asp241 carbon saturation. Shown profiles correspond to the atom given in labels. (A) C^{α} and (B) C' CEST profiles. All shown profiles correspond to the *trans* state of DREB2A. Profiles from *cis* DREB2A could not be analyzed due to poor signal to noise. Due to pulse sequence details, the C^{α} experiment was not able to probe glycine residues.

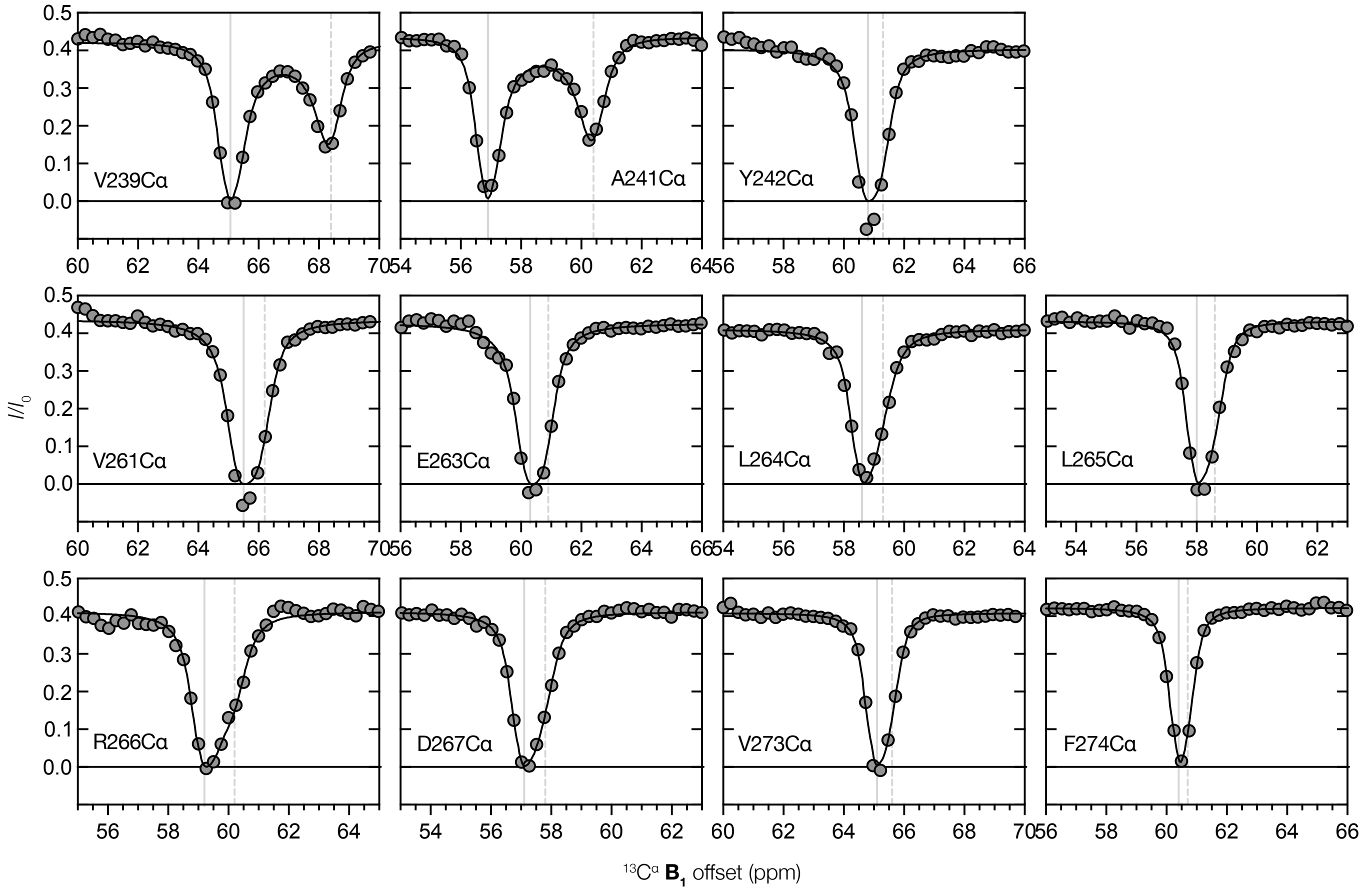

Supplementary Figure 14: ^{13}C-CEST profiles of the ABS-RIM$_{P245A}$ variant with 5% unlabeled Med25-ACID. ^{13}C-CEST profiles of both ABS and RIM fitted globally using a two-state model. The experiment was performed using a **B_1** strength of 30.0 Hz.

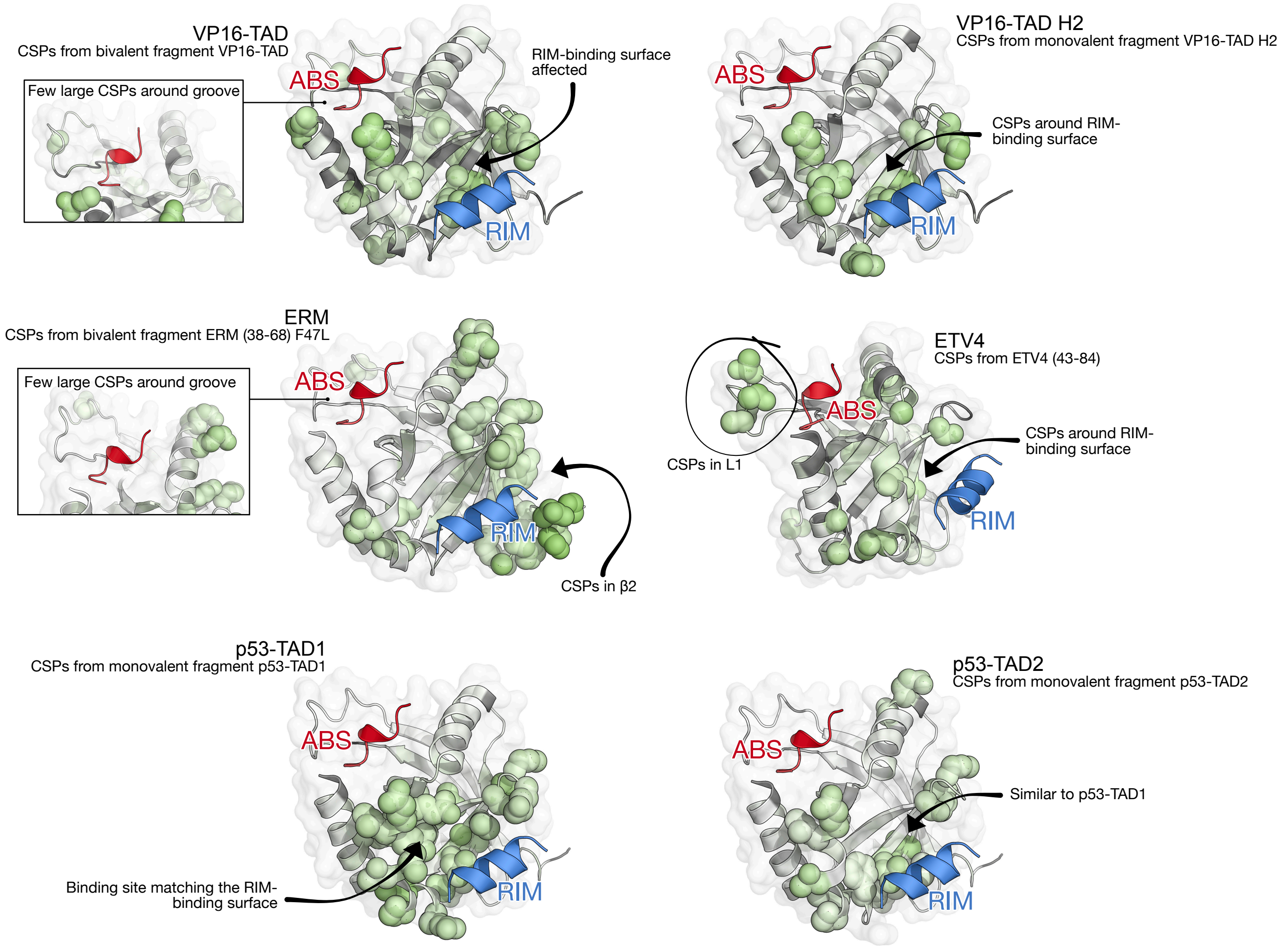

Supplementary Figure 15: *h*Med25-ACID domain binding sites superimposed with the ABS and RIM motifs from the *At*Med25-ACID:DREB2A complex model. CSPs (green) induced by VP16, ERM, ETV4 and p53 binding, mapped on the *human* Med25-ACID domain. DREB2A ABS (red) and RIM (blue) motifs from the predicted structure of the *At*Med25-ACID:DREB2A complex. Dark grey color indicates lack of data. Key observations extracted from literature[2–5] have been added to the figure.

Supplementary Method 1: Estimating affinities of *cis* and *trans* isoforms based on ^{15}N-CEST determined saturation levels. Based on NMR analysis of unbound DREB2A$_{234\text{-}276}$ (ABS-RIM) (Supplementary Fig. 10) we determined that 29.6 ± 1.3% of DREB2A is found in the *cis*-proline isoform, while 70.4 ± 1.3% is found in the *trans* isoform (Supplementary Table 1). As these populations can be considered concentrations, we calculated the equilibrium constant ($K_{\mathrm{eq,trans}\rightarrow\mathrm{cis}}$) as [*cis*DREB2A]/[*trans*DREB2A] = 0.42 ± 0.02. To determine the binding affinity of the two isoforms, we needed the solution of the two following equations:

$$K_{\mathrm{d,trans}} = \frac{[\mathrm{Med25}] \cdot [trans\mathrm{DREB2A}]}{[\mathrm{Med25}{:}\,trans\mathrm{DREB2A}]} \qquad 1$$

$$K_{\mathrm{d,cis}} = \frac{[\mathrm{Med25}] \cdot [cis\mathrm{DREB2A}]}{[\mathrm{Med25}{:}\,cis\mathrm{DREB2A}]} \qquad 2$$

From the reaction scheme (Figure 5B) we know that the concentrations of DREB2A must satisfy the following equation:

$$[\mathrm{DREB2A}]_{\mathrm{total}} = [trans\mathrm{DREB2A}] + [cis\mathrm{DREB2A}] + [\mathrm{Med25}{:}\,trans\mathrm{DREB2A}] + [\mathrm{Med25}{:}\,cis\mathrm{DREB2A}] \qquad 3$$

Since the equilibrium constant for the proline isomerization is known, we know that, at equilibrium, the concentration of free *cis*DREB2A must be:

$$[cis\mathrm{DREB2A}] = K_{\mathrm{eq,trans}\rightarrow\mathrm{cis}} \cdot [trans\mathrm{DREB2A}] \qquad 4$$

The CEST analysis revealed that 3.6 ± 0.1% of *trans*DREB2A and 6.8 ± 0.1% of *cis*DREB2A were in the bound state ($\mathrm{P_{bound}}$) and thus the concentrations of the complexes can be expressed as a function of the total concentrations:

$$[\mathrm{Med25}{:}\,trans\mathrm{DREB2A}] = \mathrm{P_{bound,trans}} \cdot ([trans\mathrm{DREB2A}] + [\mathrm{Med25}{:}\,trans\mathrm{DREB2A}]) \qquad 5$$

$$[\mathrm{Med25}{:}\,cis\mathrm{DREB2A}] = \mathrm{P_{bound,cis}} \cdot ([cis\mathrm{DREB2A}] + [\mathrm{Med25}{:}\,cis\mathrm{DREB2A}]) \qquad 6$$

These equations can be rewritten as a function of free DREB2A concentrations:

$$[\mathrm{Med25}{:}\,trans\mathrm{DREB2A}] = 3.6\% \cdot \frac{[trans\mathrm{DREB2A}]}{1 - 3.6\%} \qquad 7$$

$$[\mathrm{Med25}{:}\,cis\mathrm{DREB2A}] = 6.8\% \cdot \frac{[cis\mathrm{DREB2A}]}{1 - 6.8\%} \qquad 8$$

Applying equations 4, 7, and 8 in equation 3, the concentration of *trans*DREB2A can thus be expressed as a function of total DREB2A:

$$[\mathrm{DREB2A}]_{\mathrm{total}} = [trans\mathrm{DREB2A}] + K_{\mathrm{eq,trans}\rightarrow\mathrm{cis}} \cdot [trans\mathrm{DREB2A}] + 3.6\% \cdot \frac{[trans\mathrm{DREB2A}]}{1 - 3.6\%} + 6.8\% \cdot \frac{K_{\mathrm{eq,trans}\rightarrow\mathrm{cis}} \cdot [trans\mathrm{DREB2A}]}{1 - 6.8\%} \qquad 9$$

$$\frac{[\mathrm{DREB2A}]_{\mathrm{total}}}{[trans\mathrm{DREB2A}]} = 1 + K_{\mathrm{eq,trans}\rightarrow\mathrm{cis}} + 3.6\% \cdot \frac{1}{1 - 3.6\%} + 6.8\% \cdot \frac{K_{\mathrm{eq,trans}\rightarrow\mathrm{cis}}}{1 - 6.8\%} \qquad 10$$

$$[trans\text{DREB2A}] = \frac{[\text{DREB2A}]_{\text{total}}}{1 + K_{\text{eq,trans}\rightarrow\text{cis}} + 3.6\% \cdot \frac{1}{1 - 3.6\%} + 6.8\% \cdot \frac{K_{\text{eq,trans}\rightarrow\text{cis}}}{1 - 6.8\%}} \quad (11)$$

$$[trans\text{DREB2A}] = \frac{500\ \mu\text{M}}{1 + 0.42 + 0.038 + 0.031} = 335.9\ \mu\text{M} \quad (12)$$

Thus, we can now calculate the concentrations of the other DREB2A states.

$$[cis\text{DREB2A}] = K_{\text{eq,trans}\rightarrow\text{cis}} \cdot [trans\text{DREB2A}] = 141.0\ \mu\text{M} \quad (13)$$

$$[\text{Med25}{:}\,trans\text{DREB2A}] = 3.6\% \cdot \frac{[trans\text{DREB2A}]}{1 - 3.6\%} = 12.7\ \mu\text{M} \quad (14)$$

$$[\text{Med25}{:}\,cis\text{DREB2A}] = 6.8\% \cdot \frac{[cis\text{DREB2A}]}{1 - 6.8\%} = 10.4\ \mu\text{M} \quad (15)$$

In order to solve the equilibrium constant equations, we need the concentration of free Med25-ACID. This could theoretically be derived from the added amount of ~25 μM, however the affinity is high and thus small errors in the concentration measurements, where we expect relatively large errors, would have a massive effect on the calculated affinities. Since we have determined the overall affinity of $\text{DREB2A}_{234\text{-}276}$ using ITC, we can apply the following equilibrium to determine the expected concentration of free Med25-ACID in our sample:

$$[\text{Med25}] + [\text{DREB2A}] \rightleftharpoons [\text{Med25{:}\,DREB2A}] \quad (16)$$

$$K_{\text{d}} = \frac{[\text{Med25}] \cdot [\text{DREB2A}]}{[\text{Med25{:}\,DREB2A}]} \quad (17)$$

$$[\text{Med25}] = \frac{K_{\text{d}} \cdot [\text{Med25{:}\,DREB2A}]}{[\text{DREB2A}]} \quad (18)$$

where

$$[\text{DREB2A}] = [trans\text{DREB2A}] + [cis\text{DREB2A}] \quad (19)$$

$$[\text{Med25{:}\,DREB2A}] = [\text{Med25}{:}\,trans\text{DREB2A}] + [\text{Med25}{:}\,cis\text{DREB2A}] \quad (20)$$

By substituting concentrations using equation 19 and 20, we can now evaluate equation 18:

$$[\text{Med25}] = \frac{504\ \text{nM} \cdot (12.6\ \mu\text{M} + 10.4\ \mu\text{M})}{335.9\ \mu\text{M} + 141.0\ \mu\text{M}} = 0.024\ \mu\text{M} \quad (21)$$

Which finally enables us to solve equations 1 and 2.

$$K_{\text{d,trans}} = \frac{0.024\ \mu\text{M} \cdot 335.9\ \mu\text{M}}{12.7\ \mu\text{M}} = 640\ \text{nM} \quad (22)$$

$$K_{\text{d,cis}} = \frac{0.024\ \mu\text{M} \cdot 141.0\ \mu\text{M}}{10.4\ \mu\text{M}} = 330\ \text{nM} \quad (23)$$

Errors were obtained by evaluating the entire series of calculations 10000 times using random sampled normal distributed input variables of fraction bound percentages (P_{bound}), ITC determined affinity (K_{d}) and isomerization equilibrium constant ($K_{\text{eq,trans}\rightarrow\text{cis}}$). The calculation was repeated using parameters determined at 10 °C to enable better comparison with stopped-flow data recorded at 10 °C.

REFERENCES

1. Niklasson, M. *et al.* Comprehensive analysis of NMR data using advanced line shape fitting. *J Biomol NMR* **69**, 93–99 (2017).
2. Vojnic, E. *et al.* Structure and VP16 binding of the Mediator Med25 activator interaction domain. *Nat Struct Mol Biol* **18**, 404–410 (2011).
3. Lee, M. S., Lim, K., Lee, M. K. & Chi, S. W. Structural basis for the interaction between p53 transactivation domain and the mediator subunit MED25. *Molecules* **23**, (2018).
4. Landrieu, I. *et al.* Characterization of ERM transactivation domain binding to the ACID/PTOV domain of the Mediator subunit MED25. *Nucleic Acids Res* **43**, 7110–7121 (2015).
5. Currie, S. L. *et al.* ETV4 and AP1 Transcription Factors Form Multivalent Interactions with three Sites on the MED25 Activator-Interacting Domain. *J Mol Biol* **429**, 2975–2995 (2017).
